# Sleep Disorders in a Sample of Adopted Children: A Pilot Study

**DOI:** 10.3390/children4090077

**Published:** 2017-08-29

**Authors:** Meghna Rajaprakash, Elizabeth Kerr, Benita Friedlander, Shelly Weiss

**Affiliations:** 1 Division of Neurology, The Hospital for Sick Children, Department of Pediatrics, University of Toronto, Toronto, ON M5G 1X8, Canada; elizabeth.kerr@sickkids.ca (E.K.); benitafriedlander@gmail.com (B.F.); meghna.rajaprakash@mail.utoronto.ca (M.R.); 2 Department of Pediatrics, University of British Columbia, British Columbia Children’s Hospital, Vancouver, BC V6H 3N1, Canada

**Keywords:** adoption, children, sleep disorders, attention problems

## Abstract

Sleep disorders occur in up to 25% of children and are more prevalent in children who have attention problems and attachment issues. Research shows that foster children display sleep problems, but limited knowledge exists on sleep problems in adopted children. This pilot study aimed to identify the types of sleep disorders in adopted children and associated psychosocial factors. Parents of adopted children in Ontario, Canada, ages 2–10 years were asked to complete questionnaires evaluating demographic measures, sleep history, and the presence of behavioral problems. Insomnias and parasomnias were reported in adopted children and were associated with attention problems. This pilot study emphasizes the need for further research on the underlying factors governing the relationship between poor sleep and behavioral problems in adopted children.

## 1. Introduction

Sleep is crucial for neurocognitive functioning and child development, yet over 25% of typically developing children are reported by parents to exhibit sleep disorders [1]. Among the most commonly reported sleep disorders are insomnias, which manifest as difficulties in initiating and maintaining sleep [2]. Parasomnias are frequently reported in children [3] and include slow wave arousal disorders (sleep terrors, confusional arousals, sleepwalking) in addition to nightmares, and symptoms of restless-leg syndrome and periodic limb movements.

Sleep disruption is particularly common in early childhood possibly due to a child’s need for parental support in developing and maintaining proper sleep practices. As such, sleep at this stage is largely governed by psychosocial and family factors. In school-aged children, sleep disorders are correlated with behavioral problems such as externalizing disorders, and anxiety and depression [4,5,6,7]. Of particular significance is the fact that children who display problem behaviors, such as attention deficit hyperactivity disorder (ADHD), show sleep disturbances with prevalence rates as high as 50% [8].

Adopted children are particularly at risk for sleep disorders because of the psychosocial stressors, behavioral problems, and attachment issues they experience as they transition into a new family unit [9,10,11]. The impact on sleep can vary depending on the type of adoption. Children who are internationally adopted must transition to another country of residence and culture, whereas those that are domestically adopted (either as newborns or later in childhood) are of the same nationality and country of residence as their adoptive parents.

Furthermore, there are differences between children who are placed in foster care prior to adoption versus those who are adopted as newborns. Past research has shown that foster children are particularly prone to difficulty with sleep regulation [12]. Children are placed in foster care for different reasons, but may have experienced adverse circumstances including neglect, poor parental mental health, and sometimes maltreatment, which make them particularly vulnerable to detachment from their caregivers and self-regulation problems [13]. A recent study revealed that factors such as age at initial placement, number of placements, past experiences of sexual abuse and neglect, and level of foster parent stress were all associated with poorer sleep quality [14].

To date, limited research exists on the factors affecting sleep in adopted children. One study showed that adopted children with a history of maltreatment had poorer sleep outcomes as determined by the Children’s Sleep Habits Questionnaire (CSHQ), with disordered sleep being associated with behavioral problems [15]. Similarly, research on 240 internationally adopted children showed that more than half displayed difficulties with sleep such as problems falling asleep and sleep terrors [16]. Another study of 387 internationally adopted children showed that 48% had sleep disturbance, especially with regards to high separation anxiety at bedtime, frequent waking, and night terrors [17]. However, comparable research on the types of sleep disorders in adopted children and associated psychosocial factors is lacking.

Thus, this pilot study aimed to evaluate sleep in adopted children and possible relationships with: (a) adoption history (internationally vs. nationally adopted, number and duration of foster placements, age at time of adoption), (b) family structure (one- vs. two-parent families, parent education, siblings), and (c) behavioral issues. These factors were analyzed in relation to scores on the CSHQ [18].

In view of previous research, we predicted that sleep disorders would be prevalent in adopted children, with a greater preponderance of cases in the group who experienced foster care prior to adoption and those who are internationally adopted. We also hypothesized that sleep would be associated with many aspects of the children’s psychosocial influences, which encompasses both the child’s behavioral profile and the family structure. 

## 2. Methods

### 2.1. Participants

Participants were recruited using two different methods. Families were enrolled through face-to-face discussions while attending the Adoption Resource Exchange Conference (Toronto, Canada), which is organized by the Ontario Ministry of Child and Social Service for current and prospective adoptive parents. These families were provided the questionnaires, which were returned by mail. Further recruitment was done over six months by placing a notice about the study in the monthly bulletin of the Adoption Council of Ontario. Parents who responded were sent the questionnaires by mail and later they were provided with an online version of the questionnaires (minus the questionnaire on behavior and attention) using Research Electronic Data Capture (REDCap) methodology. All parents were invited to participate irrespective of whether they had concerns about their child’s sleep. All subjects (parents/guardians) gave their informed consent for inclusion before they participated in the study. The study was conducted in accordance with the Declaration of Helsinki and the protocol was approved by the Research Ethics Board of the Hospital for Sick Children, (No. 1000038644) University of Toronto, Toronto, Canada.

Participants were eligible for the study if they had an adopted child between the ages of two and ten years and were fluent in English. Children were excluded if they had known physical, cognitive, or mental illness (e.g., ADHD, autism), had a previously diagnosed sleep disorder, or were on medication with either a hypnotic (e.g., clonazepam) or stimulant (e.g., methylphenidate, modafanil). Parents or caregivers provided written informed consent initially, and consent by Internet prior to completing the online questionnaire. 

### 2.2. Questionnaires

#### 2.2.1. Demographics

The demographics section of the questionnaire was created for this study to obtain details on the child’s adoption history, number, and duration of placements prior to adoption, current family structure, caregiver education history (as a marker of socioeconomic status), and reported medical and behavioral problems.

#### 2.2.2. Children’s Sleep Habits Questionnaire

The CSHQ was used as the primary outcome measure in this study to separate poor sleepers and good sleepers [18]. The CSHQ is a parent-reported measure that includes a total score and eight subscale scores that screens for potential sleep disorders including both behavioral and medical. These disorders include bedtime resistance, sleep onset delay, night wakings, sleep anxiety, parasomnias, disordered breathing, daytime sleepiness, and sleep duration. A total score of greater than 41 on this scale indicates the possible presence of a sleep disorder. The Children’s Sleep Habits Questionnaire is a validated scale for identifying sleep disorders with a sensitivity of 0.80 and specificity of 0.72 [18].

#### 2.2.3. BASC-2 Measure of Behavior/Inattentive Symptoms

The Behavior Assessment System for Children, Second Edition (BASC-2) is a standardized measure assessing parent perspective of children’s internalizing, externalizing, and attention-related behavior [19]. It is a parent rating scale with separate forms for pre-school (two to five years) and child (six to ten years). The BASC-2 questions are structured in true/false format or a four-point scale that includes never, sometimes, often, and almost always. The forms are scored using computer entry, which generates T-scores and percentile ranks for each measure based on national norms.

### 2.3. Statistical Analyses

Data was analyzed with IBM SPSS Statistics 21.0 (Chicago, IL, USA). Descriptive statistics were used to describe the sample demographics. Given the non-normality of the CSHQ scores, group differences between children reported to have sleep problems vs. those that did not were analyzed using Mann-Whitney U tests. To account for multiple comparisons from the eight sleep subscales, results were corrected using the Bonferonni adjusted α level of 0.006 per test (0.05/8). Spearman’s ρ was used to calculate correlations between sleep scores and psychosocial determinants. Behavioral scores were analyzed in relation to normative data using *t*-tests.

## 3. Results

### 3.1. Demographics

Participants were parents of 48 children, ages 2.0 to 9.0 years (mean (M) = 4.5, standard deviation (SD) = 1.8). Twenty-two children were male. Mean age at adoption was 1.7 years (SD = 1.4). Mean age at time of questionnaire was 4.5 years (SD = 1.8). Twenty-two children were adopted internationally and did not differ in age from nationally adopted children. Seventy-one percent of children were in one to three foster placements prior to being adopted. Demographic factors are summarized in Table 1.

Twenty of the participants filled out paper questionnaires that were mailed out with a 67% response rate. The remaining 28 participants participated online.

### 3.2. Sleep Measures

Participants in the pre-school age category (two to five years) had a mean sleep duration of 11.34 h (SD = 1.18) while those in the school age group (six to ten years) had a mean sleep duration of 10.27 h (SD = 1.21).

**Figure 1:**
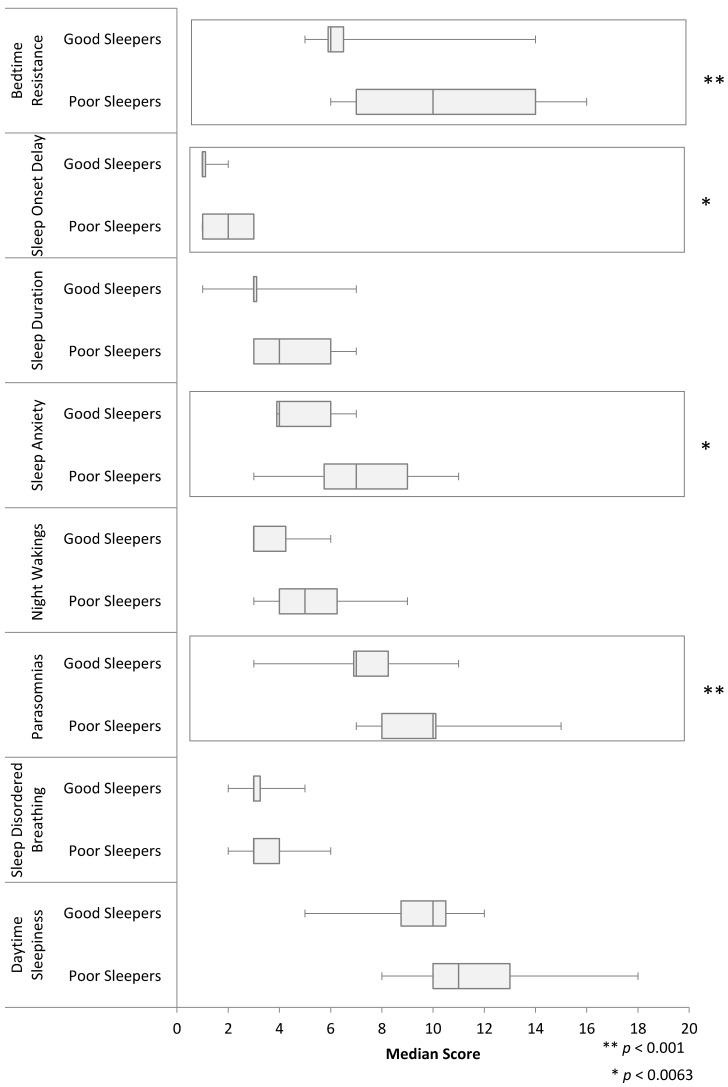
Boxplot of the Children’s Sleep Habits Questionnaire (CSHQ) scores showing the differences between good sleepers vs. poor sleepers in terms of median (vertical line), interquartile range (box where given) and whiskers to maximum and minimal values.

Mean CSHQ score was 46.2 (SD = 8.0) with 34 children (71%) having their parent endorse a possible sleep disorder (CSHQ > 41). Further analysis comparing those with and without a sleep disorder showed a significant difference in bedtime resistance, sleep onset delay, sleep anxiety, and parasomnias (*p* < 0.006). Figure 1 presents the differences in median CSHQ scores for each specific type of sleep disorder.

### 3.3. Power Analysis

The sample sizes of the two groups were 14 (no sleep disorder) and 34 (sleep disorder). Analysis of these two sample sizes achieves 100% power to detect a difference of 8.3 between the null hypothesis that both group means are 46.2 and the alternative hypothesis that the mean of the no sleep disorder group is 37.9. This includes estimated group standard deviations of 8.0 (sleep disorder group) and 2.5 (no sleep disorder group) and with a significance level (α) of 0.006 using a two-sided Mann-Whitney test, assuming that the actual distribution is double exponential.

### 3.4. Psychosocial Determinants

No significant differences in sleep scores were found between internationally and nationally adopted children, those in foster care and not in foster care, one- and two-parent families, or educated parents and those with university graduate or professionally certified caregivers. No significant relationship existed between CSHQ scores and age at questionnaire and age at adoption or length of time in adoptive care. However, several trends were noted. A difference in bedtime resistance (*p* = 0.09) was found with internationally adopted children showing higher scores (median = 11.5, Range = 6–16) than nationally adopted children (median = 7.5, Range = 5–15). In terms of family structure, a difference (*p* = 0.09) in night wakings was found with children of single parents showing higher degrees of night wakings (median = 5.0, Range = 3–9) as compared to those with two-parent families (median = 4.0, Range = 3–6).

### 3.5. Behavioural Factors

A subset of the total sample (*n* = 20) derived from the paper-based data collection was used to analyze behavioral factors. Adopted children had higher scores than normative data in hyperactivity (*p* = 0.019, mean = 57.8, SD = 13.2) with 35% rated one standard deviation or more above the mean. Higher scores than normative data were also observed on attention problems (*p* = 0.030, mean = 55.8, SD = 10.9) with 40% rated one standard deviation or more above the mean. A significant group difference was evident in attention problems (*p* = 0.015) with poor sleepers (mean = 48.3, SD = 13.2) displaying more attention problems as compared to good sleepers (mean = 59.8, SD = 7.2).

## 4. Discussion

The primary aim of this pilot study was to investigate the types of sleep disorders experienced by adopted children. The second objective was to investigate the relationship between sleep disorders and psychosocial factors including adoption history, parental education, family structure, and behavioral factors.

Results showed that the most common disorders were bedtime resistance, parasomnias, sleep onset delay, and sleep anxiety. No significant relationship existed between sleep disorders and family structure and adoption history. Finally, in the subset of the sample, adopted children had greater than average problems with attention and hyperactivity and those reporting sleep disorders showed more attention problems than good sleepers.

The pattern of sleep disorders found in the current study is consistent with past research on adopted children, which reported difficulties in falling asleep, experiencing night terrors, and not wanting to sleep alone [16]. Children reared in their biological parents’ homes also experience sleep disorders such as trouble falling asleep (23%), bedtime refusal (42%), and night terrors (7%), but these disorders occur less frequently [3]. However, in the current study on adopted children, the 71% prevalence rate is also much higher than what we predicted based on the previous study on adopted children [16] in which only half of the population was reported to have sleep disorders. This may represent increased prevalence of sleep disorders in adopted children in Ontario or may be a product of selection bias.

No relationship was found between sleep disorders and the number and duration of foster placements, type of adoption (international vs. national), or age at adoption. This may be because the adopted children in our sample were exposed to similar degrees of early childhood adversity prior to adoption. Alternatively, the stable adoptive environment may offset the effects of early adverse events on sleep in adopted children.

Family structure also did not significantly affect sleep scores. No significant difference was found between college-educated parents and those with university certifications, which can be used as a proxy for socioeconomic status. These results are consistent with a study on foster children, in which children from low-income environments did not have significantly different sleep behaviors compared to children from middle to upper income households [10]. It is possible that since none of the children in the current sample or past study were living below the poverty line, they were exposed to comparable levels of comfort in their sleep environment.

Some trends were noted in the study. First, internationally adopted children had higher levels of bedtime resistance as compared to those who were nationally adopted. As suggested previously, sleep issues are common in internationally adopted children, especially when they come from countries that practice co-sleeping and have difficulty transitioning to sleeping independently [20]. Young internationally adopted children were also found to have a high prevalence of self-regulatory dysfunction, which may explain the difficulty in settling themselves at bedtime and falling asleep by themselves [17]. Results also showed emerging trends for the association between family structure and sleep disorders. Children with single parents were found to have higher levels of night wakings as compared to those with two parents. The reason for this association is not clear but may be due to factors related to having more comfort and security with two caregivers. 

Notably, the child’s behavioral profile showed more profound effects on sleep disorders than family structure or adoption history in this study. In the subset of 20 participants, children showed higher levels of inattention-hyperactivity behaviors than typically-developing age peers despite excluding cases of known ADHD. These findings may suggest that there are cases of undiagnosed ADHD in the present sample but nonetheless points to increased attention problems in adopted children.

Further, the adopted children reported to be poor sleepers showed higher degrees of attention problems than those reported to be good sleepers. This finding is consistent with past research that documented that sleep disorders are more prevalent in children who display inattentive/hyperactive problem behaviors with prevalence rates as high as 50% [8]. Children with ADHD have more difficulty falling asleep, report restless sleep, and have shorter sleep durations [21]. The association between ADHD and sleep disturbance may be attributed to the overlapping neural underpinnings that regulate sleep and attention/arousal neurotransmitter pathways [8]. Alternatively, it has been suggested that a lack of sleep may exacerbate attention/hyperactive tendencies in children [22].

Although this study provides interesting insights into sleep disorders in adopted children, several limitations exist. First, the sample size was small, making it relatively underpowered to analyze the complex psychosocial determinants of sleep. Another limitation was selection bias, which may not have yielded a representative sample of adopted children. It is possible that parents who were most concerned about their child’s sleep disorder were more likely to participate despite the study’s inclusion of all families. However, this bias would have affected prevalence rates more so than the identification of types of sleep disorders and associated factors, which are the primary aims of the study. We also used parental assessments of child’s sleep, rather than objective analysis such as actigraphy or polysomnography, which may have further introduced bias. Finally, the CSHQ measure of sleep is not a clinical tool for diagnosing sleep disorders but rather a screen for the possible presence of sleep problems. Thus, future studies should aim to corroborate the CSHQ data with patient history and clinical diagnoses in a larger sample to best confirm the findings.

## 5. Conclusions

The current study provided preliminary evidence that adopted children experience sleep disorders, particularly with bedtime resistance, parasomnias, sleep-onset delay, and sleep anxiety. However, no significant relationship was found between sleep disorders and the family structure or adoption history. Analysis of a subset of the data revealed that parents endorsing sleep problems also noted more attention problems in their adopted children. Results suggest that healthcare workers and parents caring for adopted children should screen for sleep disorders, which may be indicative of comorbid behavioral and attention issues. Future studies should explore the risk factors contributing to the persistence of sleep disorders in adopted children.

## Figures and Tables

**Table 1:** Demographic Information.

		Number (%)
Sex	Male	22 (45.6)
	Female	26 (38.6)
Adoption type	International	22 (45.6)
	National	26 (38.6)
Family structure	Two-parent	37 (77.1)
	One-parent	11 (22.9)
Siblings	Yes	30 (52.6)
	No	18 (31.6)
Number of foster placements	None	14 (24.6)
	1–3	34 (70.8)
